# Complete Genome Sequence of Nocardia iowensis DSM 45197^T^ (= NRRL 5646^T^)

**DOI:** 10.1128/mra.00581-22

**Published:** 2023-01-04

**Authors:** Aneisha Collins Fairclough, Cathrin Spröer, Jolantha Swiderski, Boyke Bunk, Andrew S. Lamm

**Affiliations:** a Natural Products Research Laboratory, Faculty of Science and Sport, University of Technology, Kingston, Jamaica; b Leibniz Institute DSMZ-German Collection of Microorganisms and Cell Cultures, Braunschweig, Germany; c German Centre of Infection Research, Partner Site Hannover-Braunschweig, Braunschweig, Germany; Indiana University, Bloomington

## Abstract

We report the complete genome sequence and predicted functional profile of Nocardia iowensis DSM 45197^T^. N. iowensis DSM 45197^T^ is a spore-forming, mesophilic, Gram-positive bacterium that was isolated from garden soil in Osceola, Iowa, USA. This organism has been exploited for its production of glycocinnamolyspermidine antibiotics and biotransformation of xenobiotic substances. Other significant features of N. iowensis DSM 45197^T^ include the first fully characterized carboxylic acid reductase (CAR) and the first bacterial nitric oxide synthase system. The genome sequence of N. iowensis DSM 45197^T^ can facilitate further understanding of its function, as well as the pathogenesis of *Nocardia* spp. N. iowensis DSM 45197^T^ has a genome size of 8.95 Mbp; about 46% of the coding sequences have no known homologues and were labeled hypothetical proteins. This finding implies further potential for biomedical and biotechnological research applications.

## ANNOUNCEMENT

We report the complete sequencing of Nocardia iowensis DSM 45197^T^, a versatile bacterium that was initially of interest as an antibiotic producer. Subsequent discoveries of versatile biocatalytic abilities have sustained interest because it biotransforms many natural and synthetic substrates into value-added products, including flavonoids, vanillic acid, and 4-vinylphenol (1). The first fully characterized ATP/NADPH-dependent carboxylic acid reductase (CAR) was isolated from N. iowensis and has been the template for the discovery of other CAR enzymes (2–5). Many *Nocardia* spp. are pathogenic, causing opportunistic and sometimes serious infections in humans. N. iowensis DSM 45197^T^ is not known to be pathogenic, but it possesses a bacterial nitric oxide synthase system and enzymes involved in tetrahydrobiopterin biosynthesis (1), both of which are involved in susceptibility to mycobacterial infection (6).

N. iowensis DSM 45197^T^ was grown on brain heart infusion medium at 28°C. After 18 days, the late exponential growth phase was reached, and the biomass was harvested for subsequent DNA extraction. Genomic DNA extraction was carried out using the MasterPure Gram-positive DNA purification kit (Lucigen, Middleton, WI, USA) according to the manufacturer's instructions. DNA integrity was checked on a FemtoPulse system (Agilent, Santa Clara, CA, USA). A SMRTbell template library was prepared according to the instructions from Pacific Biosciences (PacBio) (Menlo Park, CA, USA) (7). Briefly, for the preparation of 10-kb libraries, 1.5 μg genomic DNA was sheared using the Megaruptor 3 (Diagenode, Denville, NJ, USA) according to the manufacturer's instructions; shearing to approximately 3 kb was performed using MagBeads. DNA was end repaired and ligated to barcoded adapters using components from the SMRTbell Express template preparation kit 2.0. Samples were pooled according to the calculations provided by the Microbial Multiplexing Calculator (https://www.pacb.com/wp-content/uploads/Express-Microbial-Multiplexing-Calculator.xlsm). Conditions for annealing of sequencing primers and binding of polymerase to purified SMRTbell templates were assessed with the calculator in single-molecule real-time (SMRT) Link. Libraries were sequenced on the Sequel *IIe* platform (PacBio), taking one 15-h movie per SMRT cell. In total, one SMRT cell resulted in 109,275 reads, with a mean (filtered) length of 7,321 bp and an *N*_50_ value of 83,86 bp. Read quality control, error correction (preassembly), and adapter trimming were performed using the PacBio microbial assembly protocol.

**FIG 1:**
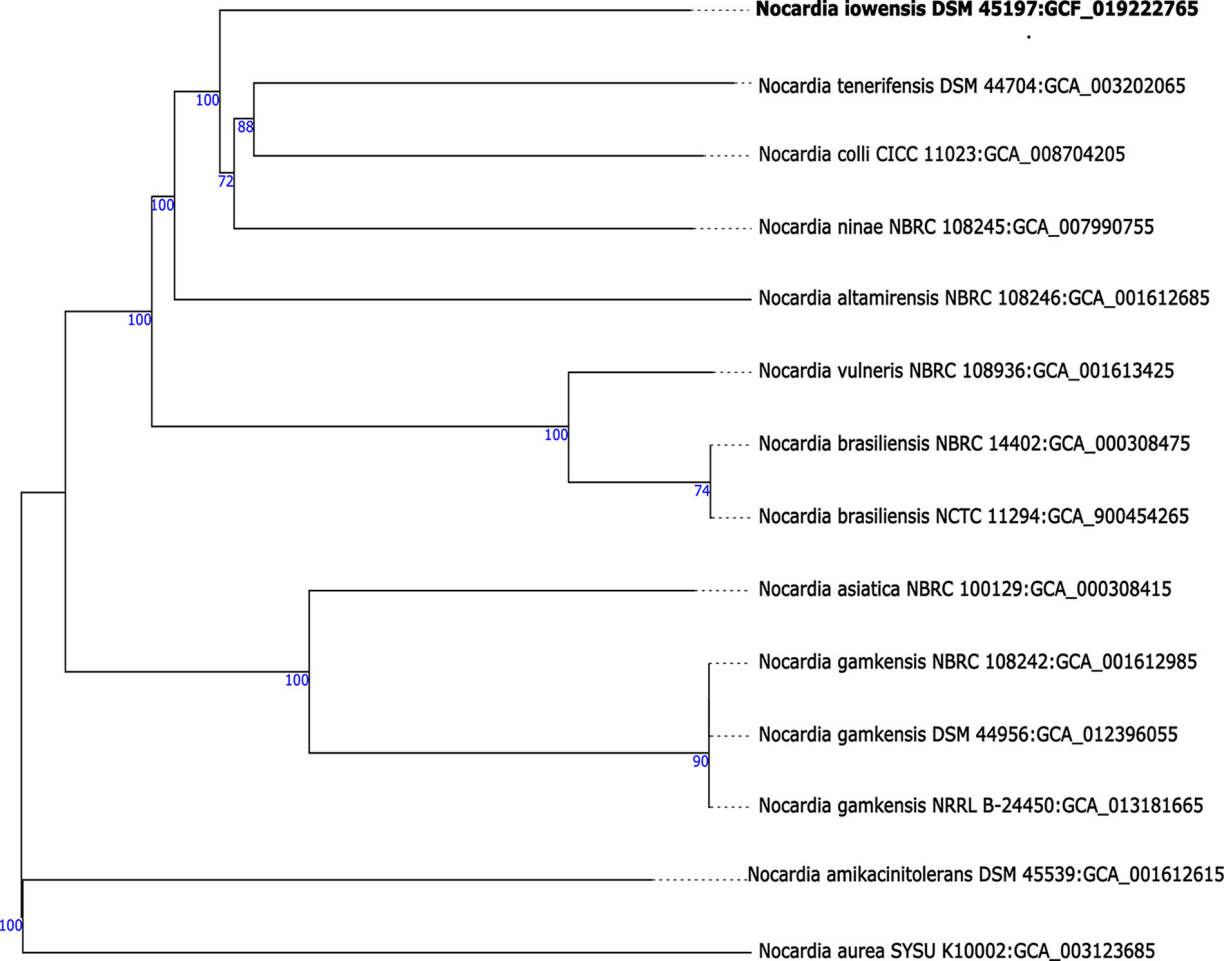
Phylogenetic tree of Nocardia iowensis DSM 45197^T^ based on *in silico* DNA-DNA hybridization genome comparisons with type strains using the Type Strain Genome Server (TYGS). GenBank assembly accession numbers follow the organism names.

The same batch of DNA was used for sequencing on the Illumina platform. A library containing inserts of 300 to 400 bp was prepared by using the Nextera XT DNA library preparation kit (Illumina, San Diego, CA, USA) with modifications (8). Samples were sequenced on a NextSeq 500 system (Illumina), which yielded 2,757,734 reads (2 × 151 bp). Quality control of Illumina reads was performed using FastQC (https://www.bioinformatics.babraham.ac.uk/projects/fastqc). Long-read genome assembly was performed using the microbial assembly protocol included in SMRT Link v10.1.0, with a target genome size of 8 Mbp. The microbial assembly process resulted in one circular chromosomal contig, which was adjusted to *dnaA*. Identification of *dnaA* was carried out using BLAST, and circularization and rotation were performed with the genomecirculator.py tool (https://github.com/boykebunk/genomefinish). Error correction was performed by mapping Illumina short reads onto the completed genome using BWA (9), with subsequent determination of a new consensus sequence (https://github.com/JHartlich/AlternateReferenceMaker). The genome was annotated using the NCBI Prokaryotic Genome Annotation Pipeline (PGAP) (Fig. 1 and Table 1). Default parameters were used for all software except where otherwise noted.

**TABLE 1:** Genomic features of Nocardia iowensis DSM 45197^T^

Feature	Finding
Length (bp)	8,948,074
Status	Complete
No. of contigs	1
GC content (%)	67.32
No. of genes	8,257
No. of coding sequences	8,191
No. of rRNAs	9
No. of tRNAs	54

### Data availability.

Complete genome sequences of Nocardia iowensis DSM 45197^T^ have been deposited in GenBank under BioProject accession number PRJNA743876, BioSample accession number SAMN20062579, SRA accession numbers SRX11360990 (PacBio reads) and SRX11360991 (Illumina reads), and GenBank nucleotide accession number CP078145.
